# Endoscopic Mini- or Less-Open Sublay Repair in a Patient with a Reduced Pulmonary Function: A Case Report

**DOI:** 10.70352/scrj.cr.26-0093

**Published:** 2026-04-24

**Authors:** Sakyo Ohashi, Tomohiro Takeda, Masahide Otani, Yasuhiro Kawashima, Tomokazu Hoshi, Yasuhiro Fujiwara, Hideki Yokoo

**Affiliations:** 1 Department of Surgery, Furano Kyokai Hospital, Furano, Hokkaido, Japan; 2 Department of Surgery, Asahikawa Medical University, Asahikawa, Hokkaido, Japan; 3 Department of Anesthesiology, Furano Kyokai Hospital, Furano, Hokkaido, Japan

**Keywords:** EMILOS, supraglottic airway device, umbilical hernia

## Abstract

**INTRODUCTION:**

Endoscopic mini- or less-open sublay (EMILOS) is a minimally invasive extraperitoneal repair technique through a small incision that allows wide mesh placement, which may reduce surgical site infection, postoperative pain, and respiratory compromise. In the present case, we performed umbilical hernia repair using EMILOS in an elderly patient with an impaired pulmonary function, and the procedure was performed safely without major postoperative respiratory complications.

**CASE PRESENTATION:**

An 86-year-old female with asthma using inhaled budesonide–formoterol and mixed ventilatory impairment presented with abdominal pain and a firm umbilical bulge. CT revealed a 38 × 36-mm umbilical hernia with an acutely irreducible small bowel. Elective repair was planned after manual reduction. Given low pulmonary reserve and anticipated airway hyperreactivity, we selected general anesthesia with a supraglottic airway device (SGA) and an EMILOS repair. Through a 3-cm skin incision caudal to the umbilical bulge, the hernia sac was dissected. The retrorectus space was developed, and a 16 × 15-cm monofilament mesh was deployed and secured. Finally, the linea alba and anterior rectus sheath were closed.

The postoperative course was generally uneventful. Discharge was delayed for social reasons, and the patient was discharged on day 14. At the 6-month follow-up, there was no evidence of any infection or recurrence.

**CONCLUSIONS:**

This case suggests that EMILOS may allow curative treatment with relatively low invasiveness in carefully selected elderly patients with impaired pulmonary function. The use of an SGA may be a reasonable option for respiratory management in this context. However, the indication for surgery should be individualized, and conclusions regarding its benefit should be interpreted with caution due to the single-case nature of this report.

## Abbreviations

ARISCAT
: Assess Respiratory Risk In Surgical Patients in Catalonia
EMILOS
: endoscopic mini- or less-open sublay
eTEP
: enhanced-view totally extraperitoneal
FEV1
: forced expiratory volume in 1 second
FVC
: forced vital capacity
IPOM
: intraperitoneal onlay mesh
MILOS
: mini- or less-open sublay
SGA
: supraglottic airway device
VC
: vital capacity

## INTRODUCTION

Primary non-incisional abdominal wall hernias are classified by the European Hernia Society as epigastric or umbilical on the midline and Spigelian or lumbar on the lateral wall.^1)^ Newer techniques, such as eTEP and EMILOS, aim to reduce invasiveness and recurrence.^2)^ EMILOS is a minimally invasive extraperitoneal repair technique involving dissection between the rectus abdominis and its posterior sheath through a small incision, allowing wide retromuscular mesh deployment. Although retrorectus dissection usually requires endoscopic extraperitoneal insufflation, it avoids intraperitoneal mesh placement. In addition, preservation of separation from the abdominal cavity is important, and if the abdominal cavity is opened accidentally, the defect should be closed immediately.^3)^ In patients with asthma or an impaired pulmonary function, tracheal intubation can provoke bronchospasm and respiratory complications, whereas an SGA causes less mucosal irritation and may reduce postoperative complications.^4,5)^ In the present case, we performed umbilical hernia repair using EMILOS with an SGA in an elderly patient with an impaired pulmonary function, and the procedure was performed safely without major postoperative respiratory complications such as pneumonia or respiratory failure.

## CASE PRESENTATION

**Fig. 1:**
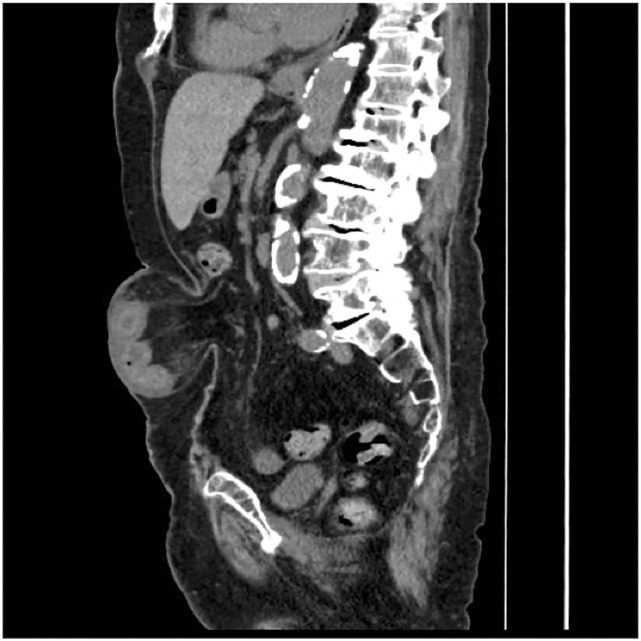
Preoperative CT (coronal view) of the abdomen. A 38 × 36-mm umbilical hernia with an acutely irreducible small bowel loop was observed.

An 86-year-old female presented with abdominal pain and a firm umbilical bulge. She had asthma treated with inhaled budesonide–formoterol (Symbicort Turbohaler; AstraZeneca, Cambridge, UK) and was a nonsmoker. She ambulated using a cane. Her vital signs were stable. Laboratory studies showed minimal inflammation, with a white blood cell count of 7280/μL and C-reactive protein level of 0.17 mg/dL. Electrocardiography and transthoracic echocardiography findings were unremarkable. CT revealed a 38 × 36-mm umbilical hernia with an acutely irreducible small bowel (**Fig. 1**). Elective repair was planned after manual reduction. Pulmonary function testing revealed reduced values: FEV1 was 0.62 L, the FEV1/FVC ratio was 46.3%, VC was 1.34 L, and %VC was 51.2%. Given the low pulmonary reserve and anticipated airway hyperreactivity, minimizing diaphragmatic elevation is therefore considered to be essential to prevent postoperative respiratory failure. Therefore, we selected general anesthesia with an SGA (i-gel supraglottic airway, size 3; Intersurgical, Wokingham, UK) and EMILOS repair. Neuraxial anesthesia was precluded in this case because of scoliosis.

**Fig. 2:**
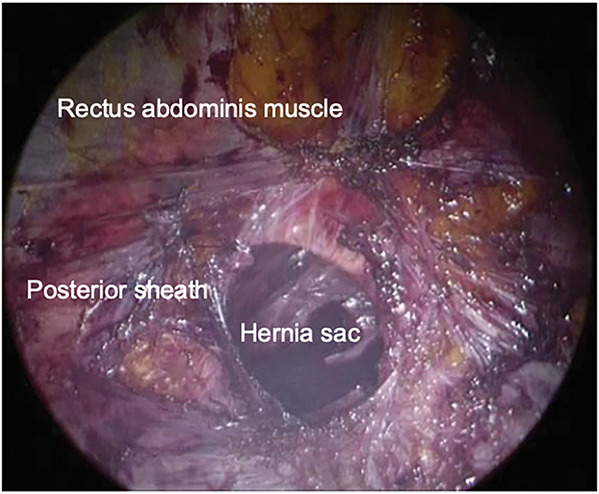
Intraoperative image after insufflation. Insufflation was established in the retrorectus space between the rectus abdominis and its posterior sheath; the hernial sac inverted toward the intraperitoneal side was visible.

**Fig. 3:**
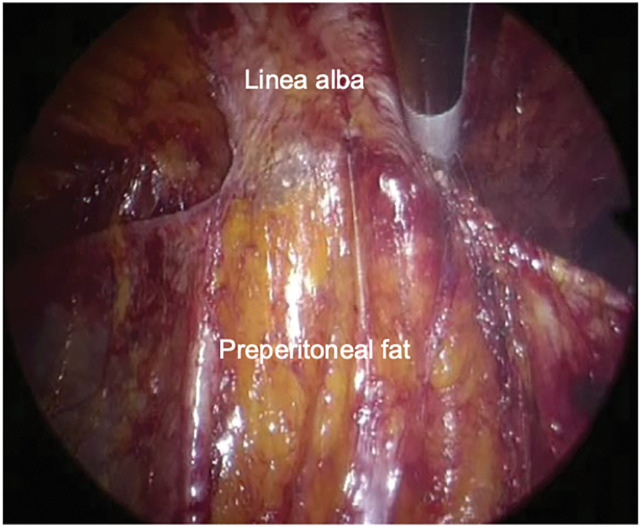
Intraoperative image after defect closure. After stopping insufflation, the peritoneum was excised.

**Fig. 4:**
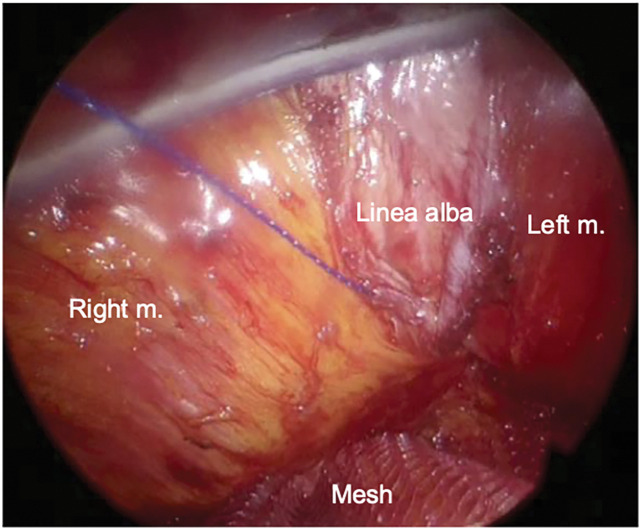
Intraoperative image during reconstruction. A 16 × 15-cm monofilament mesh was placed in the retrorectus space and secured to the peritoneal suture line. The linea alba was re-approximated to correct the diastasis with continuous nonabsorbable sutures. In this figure, “m.” denotes the rectus abdominis muscle.

**Fig. 5:**
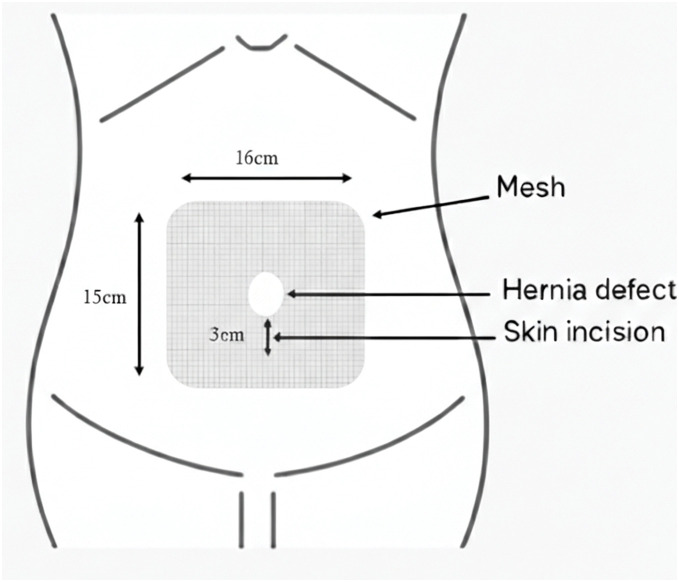
Schematic illustration of the hernia defect, skin incision and mesh placement.

Surgery was performed in the supine position under general anesthesia. A 3-cm longitudinal incision was made caudal to the umbilicus. The hernial sac was circumferentially dissected to the defect border, and the abdomen was opened. The acutely irreducible greater omentum was reduced, and the sac was temporarily closed. The rectus abdominis muscles were identified, and the anterior and posterior rectus sheaths, including the linea alba, were dissected. A single-port platform (Hakko, Nagano, Japan) was attached, and the abdominal wall was insufflated to 10 mmHg (**Fig. 2**). The retrorectus space was dissected laterally to the outer edge of the rectus abdominis to obtain sufficient mesh overlap. After stopping the insufflation, the temporary suture was released, the peritoneum was excised, and the defect was closed with 3-0 absorbable sutures (**Fig. 3**). A monofilament polyester mesh (Versatex monofilament mesh, 30 × 30 cm; Covidien, Mansfield, MA, USA) trimmed to 16 × 15 cm was implanted in the retrorectus space and secured to the peritoneal suture line with 3-0 absorbable sutures. The operative field was maintained using an abdominal wall-lifting method with muscle retractors. The linea alba was re-approximated to correct the diastasis with a continuous nonabsorbable suture (**Fig. 4**). After additional local anesthesia, the wound was closed to complete the procedure (**Fig. 5**). Immediately before removing the SGA, the airway pressure increased, and wheezing was noted. Subsequently, subcutaneous epinephrine was administered, which promptly improved the patient’s condition and thus allowed an uneventful removal of the device. The operative time was 165 min, and blood loss was 1 mL. Anesthesia was induced with fentanyl, propofol, and rocuronium. Maintenance was achieved with sevoflurane and remifentanil, with additional fentanyl and ketamine administered as needed. At emergence, neuromuscular blockade was reversed with sugammadex. Intraoperatively, SpO_2_ remained 95%–100% and EtCO_2_ 28–45 mmHg without significant fluctuation. Lung compliance was 39 mL/cmH_2_O, and the capnometer showed no abnormalities.

On the day of surgery, the patient developed a cough that rapidly improved with inhaled therapy. Postoperative analgesia was managed with continuous intravenous fentanyl infusion until POD 1. After discontinuation, the patient reported no pain, and no additional analgesics were required. Oral intake was resumed on POD 1, and her recovery was uneventful. The patient was discharged on day 14 with no complications. Although medically fit for discharge, the patient’s discharge was delayed due to social reasons. At the 6-month follow-up, there was no evidence of infection or recurrence.

## DISCUSSION

First, EMILOS may enable curative treatment with minimal invasiveness even in elderly patients with an impaired pulmonary function. In this case, when selecting the surgical approach, we kept in mind procedures that do not require intraperitoneal insufflation or can minimize it, from the perspective of minimizing the impact of intraperitoneal insufflation on respiratory mechanics in a patient with reduced respiratory reserve. MILOS may be advantageous because it can completely avoid intraperitoneal insufflation. It was also considered feasible in the present case, given the size of the hernia.^6)^ However, because the lesion was a small midline defect accessible through the hernia orifice, we considered that retrorectus dissection and wide retromuscular mesh placement could be performed more easily and reliably through the same mini-open incision under endoscopic guidance. In addition, whereas MILOS may require a dedicated device such as the Endotorch (Richard Wolf, Knittlingen, Germany), the single-incision EMILOS approach used in this case was feasible with conventional laparoscopic instruments. For these reasons, EMILOS was selected. To minimize the respiratory burden, extraperitoneal insufflation was applied only at low pressure and only during the dissection phase. Thereafter, the operative field was maintained without ongoing insufflation. No dedicated lifting system or specialized MILOS device was used. Instead, in steps performed near the hernia orifice, including closure of the defect, the field was exposed with muscle retractors, and the procedure was completed under direct vision using conventional open surgical instruments. EMILOS involves dissection between the rectus abdominis and its posterior sheath through a small incision and allows wide mesh deployment. Postoperative pain suppresses ventilation and sputum clearance, fostering atelectasis and pneumonia.^7)^ Small-incision EMILOS is expected to reduce pain, preserve deep breathing and cough, and facilitate sputum expectoration. EMILOS is a single-port technique that can be performed through the same incision as MILOS, and it can be carried out relatively easily using conventional instruments. It also has the advantage that the extent of dissection and mesh deployment can be advanced under direct vision and endoscopic assistance.^8,9)^ However, because intraperitoneal insufflation may occur during the dissection process due to peritoneal injury, insertion of a decompression port should be considered to more strictly control and manage the effects of intraperitoneal insufflation. Carbon dioxide insufflation elevates the diaphragm, decreases lung compliance, and increases postoperative pulmonary complications.^4)^ It also increases gastric regurgitation risk,^10)^ predisposing patients to aspiration and postoperative pneumonia, which can be life-threatening in compromised patients. Therefore, in EMILOS, it is important to keep the extraperitoneal insufflation pressure as low as possible. Moreover, mesh placement in the extraperitoneal plane avoids bowel contact and reduces the risk of bowel injury and adhesions.^3)^ Lower 5-year recurrence rates than with IPOM and open repair have been reported.^8)^ A circumferential overlap of at least 5 cm is recommended, and EMILOS can accommodate a mesh equal in size to or larger than that used with IPOM or open repair.^8)^

eTEP was also considered as an alternative minimally invasive extraperitoneal repair technique. However, in the present case, the hernia was a small primary midline umbilical defect that was directly accessible through the hernia orifice. Although the original EMILOS technique is not necessarily performed as a single-incision procedure, this case could be managed through a mini-open incision at the hernia site alone. Furthermore, incorporation of the initial direct-vision step of MILOS allowed the endoscopic dissection phase, including extraperitoneal insufflation, to be kept to a minimum. These features were considered advantageous over eTEP in this high-respiratory–risk patient. In larger primary hernias or incisional hernias, however, where port placement away from the defect may be required, the choice between EMILOS and eTEP may be more debatable.

On the other hand, open IPOM plus has the disadvantages of severe postoperative pain and, because the procedural sequence requires deploying and fixing the mesh intraperitoneally before fascial closure, it is prone to mesh folding or wrinkling. Above all, we considered it preferable to avoid intraperitoneal mesh placement itself.^8)^ Open preperitoneal repair tends to require a larger skin incision compared with EMILOS, and particularly in cases like the present one with a relatively small hernia orifice, the incision length may need to match the required mesh diameter. Thus, it was considered disadvantageous in terms of minimal invasiveness.^8)^ In addition, in operations involving prosthetic implantation, any increase in the risk of surgical-site infection should be avoided as much as possible.

Second, in EMILOS, respiratory management using an SGA is useful and may contribute to avoiding major postoperative pulmonary complications. In this case, the ARISCAT postoperative pulmonary complication score was 47 points, indicating a high risk, with an expected complication incidence exceeding 40%.^11,12)^ In addition, the LAS VEGAS score was 29 points, indicating a high risk.^13)^ In the present patient, only mild cough occurred, without pneumonia or respiratory failure. This suggests that a careful preoperative risk assessment, selection of surgical techniques, and airway management with minimal respiratory impact can substantially reduce the actual risk.

Regarding anesthesia management, tracheal intubation can provoke bronchospasm and increase perioperative respiratory complications in patients with an impaired pulmonary function, such as asthma. Patients with severely impaired respiratory function should, whenever possible, avoid general anesthesia. In the present case, however, neuraxial anesthesia was considered difficult because of scoliosis. Local anesthesia was also considered unsuitable. It was judged unlikely to provide sufficient analgesia and adequate operative conditions for extensive retromuscular dissection, defect closure, and mesh placement. Therefore, general anesthesia was selected. An SGA was used to minimize airway stimulation. SGA application avoids tracheal mucosal stimulation and is associated with lower rates of postoperative cough and laryngospasm than intubation,^5)^ making it reasonable for airway hyperresponsiveness. In the present case, the occurrence of an exacerbation of asthma requiring epinephrine suggested that the patient’s asthma was severe. The patient might have been at risk of developing serious respiratory complications with tracheal intubation. Furthermore, there have been reports of sugammadex-associated bronchospasm and anaphylaxis.^14)^ In this case, because sugammadex was administered at the time of SGA removal, the possibility cannot be ruled out. Therefore, in patients with asthma, respiratory status should be monitored carefully after administration.

As this was a single case, the favorable outcome cannot be attributed to any specific single factor. Instead, it was likely the result of multiple contributing factors. In particular, the anesthetic strategy using an SGA to reduce airway stimulation, the minimally invasive retromuscular repair achieved with EMILOS, and the relatively short operative time may have contributed to the favorable postoperative course. However, larger multicenter studies are needed to confirm the efficacy and safety in patients with an impaired pulmonary function. As a single case, this report cannot support universal conclusions, and SGA use warrants caution in patients at a high risk of gastric regurgitation and in prolonged procedures. In addition, the follow-up period in this report was 6 months, which is short for the evaluation of hernia recurrence. Because low long-term recurrence has been reported as one of the advantages of EMILOS, long-term follow-up should be continued to evaluate recurrence and late outcomes such as chronic pain.

## CONCLUSIONS

In conclusion, this case suggests that EMILOS may allow curative treatment with relatively low invasiveness in carefully selected elderly patients with impaired pulmonary function. The use of an SGA may be a reasonable option for respiratory management in this context. However, the indication for surgery should be individualized, and conclusions regarding its benefit should be interpreted with caution due to the single-case nature of this report.

## DECLARATIONS

### Availability of data and materials

Data will be made available on reasonable request.
